# Propensity Score-Matched Analysis of Perioperative Outcomes of Supine versus Prone Percutaneous Nephrolithotomy

**DOI:** 10.3390/jcm13092492

**Published:** 2024-04-24

**Authors:** Roi Babaoff, Gherman Creiderman, Abd Elhalim Darawsha, Yaron Ehrlich, Bhaskar Somani, David A. Lifshitz

**Affiliations:** 1 Department of Urology, Rabin Medical Center, Petah Tikva 4937213, Israel; creiderman_g@yahoo.com (G.C.); davlifshi@gmail.com (D.A.L.); 2 Sackler School of Medicine, Tel Aviv University, Tel Aviv 6997801, Israel; 3 University Hospital Southampton NHS Foundation Trust, Southampton SO16 6YD, UK; stoneurol@gmail.com

**Keywords:** percutaneous nephrolithotomy, prone, supine, match, propensity score

## Abstract

**Objective:** To compare the perioperative outcomes of supine and prone percutaneous nephrolithotomy (PCNL). **Methods:** A retrospective search of a tertiary medical center database yielded 517 patients who underwent supine (*n* = 91) or prone (*n* = 426) PCNL between September 2015 and July 2020. Data on demographics, baseline clinical parameters, and stone burden were included as predictors in a logistic regression model, generating a set of propensity scores. Seventy patients after supine PCNL were propensity score-matched 1:1 with patients after prone PCNL and compared for operative time, perioperative complications, system complexity, and stone-free rate. **Results:** We found that the operative time was significantly shorter in the supine PCNL group than in the prone PCNL group (85.5 ± 25.2 min vs. 96.4 ± 25.8 min, respectively; *p* = 0.012). The majority of both groups had low-grade (I–II) complexity systems (85.6% and 88.6%, respectively), with no significant difference among all grade groups (*p* = 0.749). There were no significant differences between the supine and prone PCNL groups in terms of the overall perioperative complication rate (8.6% vs. 4.3%, respectively; *p* = 0.301) or stone-free rate (74.3 vs. 65.7%, respectively; *p* = 0.356), while the rate of blood transfusion was significantly higher in the supine group (*p* = 0.023). **Conclusions:** In our study, we used propensity score matching to compare patients who underwent PCNL in the supine or prone position, adjusting for selection bias. Supine PCNL was associated with a shorter operative time but a higher blood transfusion rate, with no differences in the overall complication and stone-free rates.

## 1. Introduction

Percutaneous nephrolithotomy (PCNL) is the preferred method of treating patients with a large or complex kidney stone burden. After its conception in the late 1970s, PCNL, which was traditionally performed in the prone position, rapidly gained worldwide popularity as an alternative to open surgery [1,2]. The supine technique was introduced a decade later and offered the advantages of easier anesthetic care of the patient, a more comfortable position, reduced X-ray exposure for the surgeon, and lower intrarenal pressure during surgery [3]. The easy retrograde access made it possible to combine supine PCNL with retrograde intrarenal surgery (RIRS) on the ipsilateral or contralateral kidney, such as endoscopic combined intrarenal surgery (ECIRS) or simultaneous bilateral endoscopic surgery (SBES) [4].

The prone and supine positions are considered equally safe, but neither has a proven superiority in terms of operating time, rate of complications, or stone-free rate [5,6]. The Percutaneous Nephrolithotomy Global Study from the Clinical Research Office of the Endourological Society (CROES) demonstrated a shorter mean operative time and a higher rate of complications for the prone position [7], whereas others reported a shorter operative time for the supine position [8]. However, in many of the relevant studies, including the CROES study, the comparison between the prone and supine techniques was limited because it was based on outcome results from centers that perform solely one or the other. Accordingly, some of the surgeons were not versed in both techniques. This could bias research seeking to highlight possible differences between the two.

We started performing supine PCNL in our institution in 2015. We chose less complex cases for the supine approach, based on the European Association of Urology guidelines suggesting that the “prone position offers more options for puncture and is therefore preferred for upper pole or multiple accesses”, while continuing to perform prone PCNL for various indications. Consequently, our supine PCNL patients were less likely to have complex, large-volume, or multiple-location stones that involve the upper pole. The present study aimed to compare the clinical perioperative results of supine and prone PCNL in a single-center, single-surgeon cohort. To avoid the inherited selection bias in the supine PCNL cohort, we employed propensity score matching analyses, taking individual patient baseline and clinical parameters into account before matching based on the propensity score as a balancing score. This allowed us to compare the outcomes of supine and prone PCNL between the two groups.

## 2. Materials and Methods

### 2.1. Patient Selection

**Figure 1:**
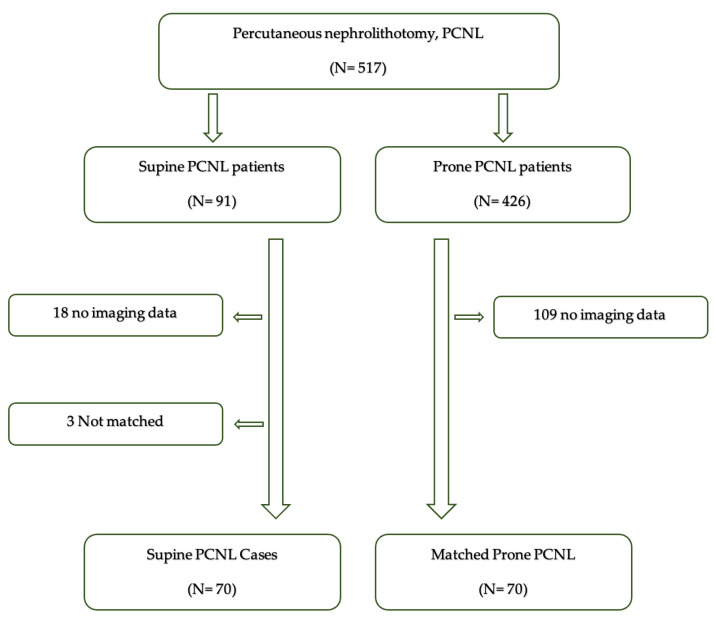
Inclusion flowchart for matching.

A retrospective search of the electronic healthcare database at Rabin Medical Center, a tertiary medical center in Petah Tikva, Israel, yielded 517 patients who underwent supine (*n* = 91) or prone (426) PCNL between September 2015 and July 2020. Demographic, clinical, and radiological parameters were collected from the medical files and Picture Archiving and Communication System (PACS). Eighteen of the ninety-one patients after supine PCNL were excluded from the analysis because their preoperative computed tomography (CT) scans were unavailable. Propensity scores were calculated for each patient based on their individual demographic, clinical, and radiological parameters. Seventy patients treated with supine PCNL were propensity score-matched 1:1 with patients treated with prone PCNL, for a total of 140 patients who formed the final cohort (Figure 1). The supine and prone PCNL groups were compared for surgical and radiological outcome measures.

All surgical procedures were performed by a single, fellowship-trained surgeon with extensive experience in endourology (D.L.). In general, the supine approach was considered for less complex cases. In addition, as most of the procedures were carried out in our endosuite (Dornier Gemini™, Wessling, Germany), the choice of position was also based on the laterality of the stone, as it is ergonomically difficult to perform supine PCNL from the same side as the fixed C-arm. Therefore, often less complex cases that would be suitable for a supine approach were actually performed in the prone position due to the operating room restrictions. All data were collected from our institutional review board-approved data registry.

### 2.2. Surgical Techniques

Preoperatively, all patients underwent a full evaluation, including a complete blood count, coagulation profile, renal function tests, urine analysis, and urine culture. A positive urine culture warranted sensitivity-specific antibiotic therapy and a repeated, negative culture preoperatively. Non-contrast CT was performed in all patients.

Prone PCNL was performed as previously described by our team [9]. The tract was placed by the surgeon except for four patients who presented for surgery with a pre-placed nephrostomy tube. The standard prone, X-ray-guided PCNL technique was utilized. Supine PCNL was performed with the patient in Giusti’s position, and the tract was placed under a combination of X-ray and ultrasound guidance. The caliceal puncture site allowing maximal stone clearance through a single tract was chosen.

In most cases, a 24 Fr balloon dilator (Nephromax™; Boston Scientific, Natick, MA, USA) was used for tract dilatation. A 20.8 rigid universal nephroscope (Richard Wolf, Knittlingen, Germany) was utilized, mostly using the inner 18 Fr scope without the outside sheath. When the stone was reached, it was fragmented with either ultrasound (ShockPuls-SE™ Lithotriper, Olympus, Tokyo, Japan) or ballistic lithotripsy (Lithospec™, Mediaspec, Gaithersburg, MD, USA). In all patients, flexible nephroscopy was used to find and treat residual stones, utilizing laser lithotripsy when required.

After the procedure, either a 6 Fr JJ stent (Percuflex™; Boston Scientific, Marlborough, MA, USA) (with a thread in the supine position) or an 8 Fr nephrostomy tube (Cook^®^-Cope Loop Nephrostomy Set, Cook Medical, Bloomington, IN, USA) was placed, according to the surgeon’s preference. A subcutaneous local anesthetic was administered at the incision site. Imaging was performed on postoperative day 1: abdominal X-ray imaging for radiopaque stones and non-contrast CT for radiolucent stones. The nephrostomy tubes were removed on postoperative day 2, and the JJ stents were removed 1–2 weeks after surgery. Follow-ups included scheduled visits to our endourology clinic (see Section 2.3).

### 2.3. Measurements and Outcomes

Preoperative surgical parameters included renal anatomy, stone location, stone burden (in mm^2^) [10], and complexity score, evaluated according to Guy’s stone score [11] by a single endourology fellow. Preoperative renal function was evaluated using the Modification of Diet in Renal Disease equation. Postoperative changes in the hemoglobin and hematocrit levels were based on the preoperative baseline values and the postoperative same-day blood test results. Postoperative complications included fever, urosepsis, administration of blood products, pleural effusion, and collecting system perforation. The stone-free rates were defined with and without residual (≤2 mm) fragments based on non-contrast CT imaging or ultrasound plus abdominopelvic X-ray imaging at the first clinic visit 4–6 weeks after surgery.

### 2.4. Statistical Analysis

The propensity score, defined in our study as the calculated probability of a patient undergoing supine PCNL, was generated using multivariate logistic regression based on the known covariates of the outcomes at baseline. We included age, gender, body mass index (BMI), estimated glomerular filtration rate (GFR), and renal stone burden as covariates. Propensity score matching was performed 1:1 (without replacement) according to the values generated for patients who underwent supine PCNL. The minimal propensity score difference for matching between the supine and prone PCNL values was based on nearest-neighbor matching within a propensity score-based caliper width of 0.2 times the logit of the propensity score. To assess propensity score matching quality, the absolute standardized mean difference was calculated for each baseline factor.

Post-matching, perioperative patient parameters, and outcomes were compared between groups using a *t*-test and Mann–Whitney U test for continuous variables and a chi-squared test for categorical variables. Conditional logistic regression was used to evaluate the association between the outcome and surgical position for the propensity-matched pairs. A two-tailed *p* < 0.05 was considered statistically significant for this study. All statistical analyses were performed using SPSS version 23.0 (IBM Corp., Armonk, NY, USA).

## 3. Results

**Figure 2:**
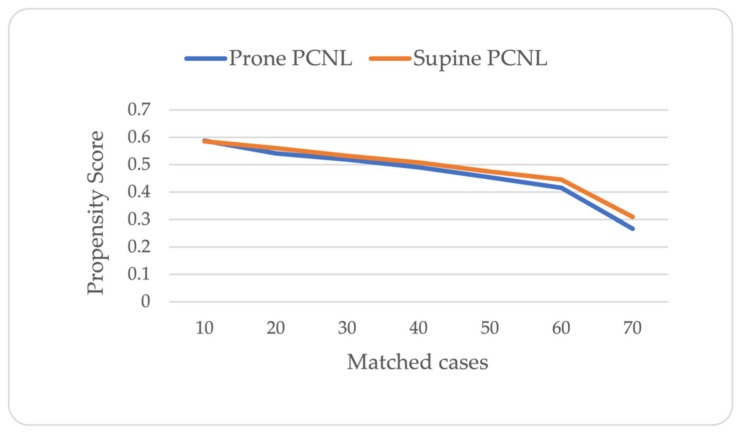
Distribution of propensity scores by treatment group.

A total of 140 patients who underwent supine or prone PCNL were matched 1:1 according to their propensity scores. Figure 2 shows the distribution of propensity scores by treatment groups. Table 1 shows the baseline and preoperative clinical characteristics of the study cohort after propensity score matching. There were no significant differences between the supine and prone PCNL groups in terms of their mean age (54.3 ± 14 vs. 55.5 ± 13.8 years, respectively), proportion of males (43 vs. 39%, respectively), mean BMI (29 ± 5.4 vs. 28.6 ± 6.6 kg/m^2^, respectively), mean GFR (109.2 ± 39.8 vs. 111.0 ± 33.7 mL/min, respectively) and left side procedure rates (67.1% vs. 54.3%, respectively). Most patients in both the supine and prone PCNL groups had no history of undergoing a prior surgical intervention (51 (73%) vs. 52 (74%), respectively; *p* = 0.848). Of the remaining cases, previous extracorporeal shock wave lithotripsy (ESWL) was performed in six (8.6%) vs. eight (11.5%) patients, and previous PCNL was performed in nine (12.8%) vs. ten (14.2%) patients in the supine and prone PCNL, respectively. Patients who underwent prone PCNL had a slightly higher stone burden (266.0 ± 145.2 vs. 239.6 ± 116.5 mm^2^, respectively), but the difference did not reach statistical significance (*p* = 0.237).

Regarding the post-propensity score matching analysis, the majority of both the supine and prone PCNL groups had a low-grade (I or II) complexity system (85.6% and 88.6%, respectively), with no significant difference among all grade groups (*p* = 0.749) (Table 2). A supracostal access was utilized more often in the prone group (32.9% vs. 14.3%, respectively; *p* = 0.016).

Within the propensity score-matched cohort, the supine PCNL group had a significantly shorter operative time (85.5 ± 25.2 vs. 96.4 ± 25.8 min, respectively; *p* = 0.012). The supine PCNL group had a larger postoperative decrease in hemoglobin level (1.7 ± 1.5 vs. 1.2 ± 1.1 g/dL, respectively; *p* = 0.026), but there was no significant between-group difference in change in hematocrit levels (5.6 ± 4.2 vs. 4.4 ± 3.8%, respectively; *p* = 0.063).

Overall, the perioperative complication rate was 6.4%. Complications included fever (>38.2 °C) and the need for blood transfusions. No other PCNL-related complications, such as urosepsis, collecting system perforation, and pleural effusion, were observed in this cohort. While there was no statistically significant difference in the overall complication rate (8.6% vs. 4.3% in the supine and prone PCNL groups, respectively; *p* = 0.301), blood transfusion was noted only in the supine group (*p* = 0.023).

The evaluation of stone-free rates at follow-up was based on CT imaging in most cases (54 (77.1%) vs. 59 (84.3%), respectively; *p* = 0.284) in the supine and prone PCNL groups, and was comparable between the two groups.

The overall stone-free rate, including minute fragments <2 mm, was 80.7% and similar between the groups. After excluding patients with residual fragments <2 mm, it decreased to 74.3% and 65.7% in the supine and prone groups, respectively (*p* = 0.268) (Table 3).

There was no association in conditional logistic regression between surgical position and the stone-free rate, calculated with or without residual fragments (Table 4).

## 4. Discussion

Although supine PCNL is a well-accepted and widespread technique, most PCNL procedures worldwide, including in our center, are still performed in the prone position [12].

According to the CROES study, based on the largest PCNL database, patients who underwent prone PCNL experienced higher rates of complications, including the need for blood transfusions and fever, than patients after supine PCNL [7]. The same results were shown for Clavien grade >2 complications in a recent multicenter randomized control study of complex cases [8]. A large meta-analysis of 12 randomized controlled studies also reported fewer infectious complications for supine PCNL [13].

In contrast to the above-mentioned studies, in which most of the participating centers predominantly used only one of the two techniques, the present study was performed in a single institution, where both techniques were performed by the same surgeon. To improve our observations and avoid selection bias, we conducted a propensity score-matched analysis of patients operated in the supine or prone position.

The overall complication rate in our study for both groups was very low (6.4%). This result was not unexpected, given that 86% of our patients had low stone complexity scores (Guy’s grade I–II) [11].

We observed only Clavien grade 1 and 2 complications (e.g., fever and administration of blood transfusions) [14]. Interestingly, we found a significant difference in the blood transfusion rates required only in the supine group, which showed a more significant drop in hemoglobin level, although the change in hematocrit level compared to the prone PCNL group did not reach statistical significance. Soma and Elshal [15] and Falahatkar and co-workers [16] also reported higher odds (by 2.5–3-fold) for transfusion in supine PCNL [15,16], whereas Perrella et al. [8], in a more recent study, reported no difference in transfusion rate between their groups. However, Perrella et al. [8] considered only refractory hypovolemia as an indicator for blood transfusion, whereas in our center, the indications were broader, including a relative drop in hemoglobin in patients with chronic ischemic heart disease (in order to prevent an exacerbation of ischemia). We hypothesize that the mobility of the kidneys in the supine position and the longer tract allow more space in the retroperitoneum for blood to accumulate and a less effective tamponade mechanism. Nevertheless, the clinical significance of this potential mechanism seems to be negligible.

Exploring the clinical implications of our findings and possible preventive measures in light of the current medical literature, the utilization of the mini PCNL technique, which has been reported to reduce blood loss and transfusion rates [17], might eventually eliminate the disparities between supine and prone PCNL. Mini PCNL may be particularly suitable for the type of patients in the current cohort with a relatively smaller stone burden.

The operative time was shorter by means of 11 min for the supine PCNL when compared to the prone PCNL, and this difference was statistically significant. A shorter operative time for supine PCNL was observed in many studies and was commonly attributed to the elimination of the need to reposition the patient [18,19]. Although supine PCNL had a significantly shorter operative time in our study, and considering further gains such as shorter anesthetic time, better lung ventilation, lower cardiovascular stress, and intrarenal pressure [3], we did not observe an effect on intraoperative patient safety, as there was no significant difference in the overall complication rate between the two techniques. A 10-minute difference should not be the sole criterion dictating the choice of technique; however, the influence of supine PCNL’s shorter operative time on recovery and healthcare resources should be explored in future, larger clinical trials.

No difference was found in the stone-free rate between the supine and prone approaches. The overall stone-free rate was 81% when residual fragments ≤2 mm were included in the analysis and 70% when they were not. These values are high compared to the study by Perrela et al. [8], which specifically only included patients with a Guys score of three and four, but similar to those reported in the recent meta-analysis by Keller et al. [13]. Clearly, the prevalence of low-complexity cases in both groups in comparison to the complex cases in Perrela’s study explains the difference. However, we agree with the conclusions in the study by Perrela et al. that both positions offer comparable results. An exception, perhaps, would be when supracostal access is planned due to factors such as a large and complex stone burden or specific renal anatomy. In the prone position, supracostal access allows an optimal percutaneous tract that is parallel to the longitudinal axis of the kidney as it starts just parallel to the supraspinal muscles aiming towards the lower pole. While studies have investigated means to reduce potential thoracic complications in the prone position [20], such data are lacking for the supine PCNL position.

This study was limited by the small sample size and retrospective study design. Most patients had low-complexity scores, but the results were in line with other series, also those including patients with high-complexity scores [8]. Furthermore, although all patients underwent preoperative CT, only in some was a combination of abdominopelvic X-ray and ultrasound used to assess the stone-free rate. Therefore, the actual stone-free rate may be lower than calculated. Notwithstanding, the same follow-up imaging protocol was used in both groups, thereby eliminating possible bias. As detailed in the CROES study [7], CT scans are only used to assess stone-free rates in about 50% of patients.

## 5. Conclusions

After adjusting for potential treatment selection biases, this study found that supine PCNL was associated with a shorter operative time than prone PCNL, with no significant difference between the two techniques in the overall complication rate and stone-free rate. However, the rate of bleeding requiring blood transfusion was higher in the supine group.

## Figures and Tables

**Table 1:** Propensity score-matched cohorts.

Variable	Supine PCNL (*n* = 70)	Prone PCNL (*n* = 70)	Total Cohort (*n* = 140)	*p*-Value
Age (yr), mean ± SD	54.3 ± 14.0	55.5 ± 13.8	54.9 ± 13.9	0.618
Gender, *n* (%)				0.493
Male	43 (52.4)	39 (47.6)	82 (58.7)	
Female	27 (46.6)	31 (53.4)	58 (41.42)	
BMI (Kg/m^2^), mean ± SD	29.0 ± 5.4	28.6 ± 6.6	28.8 ± 6.0	0.674
GFR (mL/min), mean ± SD	109.2 ± 39.8	111.0 ± 13.7	110.1 ± 36.6	0.773
Stone burden (mm^2^), mean ± SD	239.6 ± 116.5	266.0 ± 145.2	252.8 ± 131.4	0.237
Side (left), *n* (%)	47 (67.1)	38 (54.3)	85 (60.7)	0.166

SD: standard deviation; BMI: body mass index; and GFR: glomerular filtration rate.

**Table 2:** Complexity grading analysis according to Guy’s stone score.

Stone Score	Supine PCNL (*n* = 70)	Prone PCNL (*n* = 70)	Total Cohort (*n* = 140)	*p*-Value
Grade I, *n* (%)	30 (42.8)	33 (47.2)	63 (45)	0.749
Grade II, *n* (%)	30 (42.9)	29 (41.4)	59 (42.1)	
Grade III, *n* (%)	9 (12.8	8 (11.4)	17 (12.1)	
Grade IV, *n* (%)	1 (1.4)	0	1 (0.7)	

**Table 3:** Perioperative outcome covariates.

Covariate	Supine PCNL (*n* = 70)	Prone PCNL (*n* = 70)	Total Cohort (*n* = 140)	*p*-Value
Operative time (min), mean ± SD	85.5 ± 25.2	96.4 ± 25.8	91.0 ± 26.0	0.012
Change in hemoglobin (g/dl), mean ± SD	1.7 ± 1.5	1.2 ± 1.1	1.4 ± 1.3	0.026
Change in hematocrit (%) mean ± SD	5.6 ± 4.2	4.4 ± 3.8	5.0 ± 4.0	0.063
Infracostal percutaneous access, *n* (%)	60 (85.7)	47 (67.1)	107 (76.4)	0.016
Complications, *n* (%)	6 (8.6)	3 (4.3)	9 (6.4)	0.301
Fever	1 (1.4)	3 (4.3)	4 (2.9)	0.310
Need for blood transfusion	5 (7.1)	Nil	5 (3.6)	0.023
Stone-free rate, *n* (%)	52 (74.3)	46 (65.7)	98 (70.0)	0.268
Stone-free rate with residual (≤2 mm) fragments, *n* (%)	57 (81.4)	56 (80.0)	113 (80.7)	0.830

**Table 4:** Stone-free rate odds ratio.

Outcome Measure	Odds Ratio	95% Confidence Interval	*p*-Value
Stone-free rate	0.88	0.39–1.91	0.855
Stone-free rate with residual (≤2 mm) fragments	1.10	0.42–2.88	1.000

## Data Availability

Data obtained for this study are available from the corresponding author upon institutional approval (Rabin Medical Center, Petah Tikva, Israel) of a reasonable request.

## References

[1] Fernström I., Johansson B. (1976). Percutaneous pyelolithotomy. Scand. J. Urol. Nephrol..

[2] Mak D.K.-C., Smith Y., Buchholz N., El-Husseiny T. (2016). What is better in percutaneous nephrolithotomy—Prone or supine? A systematic review. Arab. J. Urol..

[3] Uria J.G.V., Gerhold J.V., Lopez J.A.L., Rodriguez S.V., Navarro C.A., Fabian M.R., Bazalo J.M.R., Elipe M.A.S. (1998). Technique and complications of percutaneous nephroscopy: Experience with 557 patients in the supine position. J. Urol..

[4] Giusti G., Proietti S., Pasin L., Casiraghi G.M., Gadda G.M., Rosso M., Kinzikeeva E., Doizi S., Gaboardi F., Traxer O. (2016). Simultaneous bilateral endoscopic manipulation for bilateral renal stones. Urology.

[5] Vicentini F.C., Mazzucchi E., Gökçe M.I., Sofer M., Tanidir Y., Sener T.E., Melo P.A.d.S., Eisner B.H., Batter T.H., Chi T. (2021). Percutaneous nephrolithotomy in horseshoe kidneys: Results of a multicentric study. J. Endourol..

[6] Melo P.A.D.S., Vicentini F.C., Perrella R., Murta C.B., Claro J.F.D.A. (2019). Comparative study of percutaneous nephrolithotomy performed in the traditional prone position and in three different supine positions. Int. Braz. J. Urol..

[7] Valdivia J.G., Scarpa R.M., Duvdevani M., Gross A.J., Nadler R.B., Nutahara K., de la Rosette J.J.M.C.H., on behalf of the CROES PCNL Study Group (2011). Supine versus prone position during percutaneous nephrolithotomy: A report from the Clinical Research Office of the Endourological Society Percutaneous Nephrolithotomy Global Study. J. Endourol..

[8] Perrella R., Vicentini F.C., Paro E.D., Torricelli F.C.M., Marchini G.S., Danilovic A., Batagello C.A., Mota P.K.V., Ferreira D.B., Cohen D.J. (2022). Supine versus prone percutaneous nephrolithotomy for complex stones: A multicenter randomized controlled trial. J. Urol..

[9] Shoshany O., Margel D., Finz C., Ben-Yehuda O., Livne P.M., Holand R., Lifshitz D. (2015). Percutaneous nephrolithotomy for infection stones: What is the risk for postoperative sepsis? A retrospective cohort study. Urolithiasis.

[10] Smith A., Averch T.D., Shahrour K., Opondo D., Daels F.P., Labate G., Turna B., de la Rosette J.J.M.C.H., CROES PCNL Study Group (2013). A nephrolithometric nomogram to predict treatment success of percutaneous nephrolithotomy. J. Urol..

[11] Thomas K., Smith N.C., Hegarty N., Glass J.M. (2011). The Guy’s stone score—Grading the complexity of percutaneous nephrolithotomy procedures. Urology.

[12] Kamphuis G.M., Baard J., Westendarp M., De La Rosette J.J.M.C.H. (2015). Lessons learned from the CROES percutaneous nephrolithotomy global study. World J. Urol..

[13] Keller E.X., DE Coninck V., Proietti S., Talso M., Emiliani E., Ploumidis A., Mantica G., Somani B., Traxer O., Scarpa R.M. (2021). European Association of Urology—European Society of Residents in Urology (EAU-ESRU). Prone versus supine percutaneous nephrolithotomy: A systematic review and meta-analysis of current literature. Minerva Urol. Nephrol..

[14] de la Rosette J.J., Opondo D., Daels F.P.J., Giusti G., Serrano A., Kandasami S.V., Wolf J.S., Grabe M., on behalf of the CROES PCNL Study Group (2012). Categorisation of complications and validation of the Clavien score for percutaneous nephrolithotomy. Eur. Urol..

[15] Shoma A.M., Elshal A.M. (2012). Nephrostomy tube placement after percutaneous nephrolithotomy: Critical evaluation through a prospective randomized study. Urology.

[16] Falahatkar S., Moghaddam K.G., Kazemnezhad E., Farzan A., Aval H.B., Ghasemi A., Shahab E., Esmaeili S.S., Motiee R., Langroodi S.A.M. (2015). Factors affecting complications according to the modified Clavien classification in complete supine percutaneous nephrolithotomy. Can. Urol. Assoc. J..

[17] EAU Guidelines Edn. Presented at the EAU Annual Congress Paris 2024. ISBN 978-94-92671-23-3. https://uroweb.org/guidelines/urolithiasis/chapter/guidelines.

[18] Miçooğullari U., Kamaci D., Yildizhan M., Kiliç F.U., Çetin T., Çakici U., Keske M., Yalçin M.Y., Ardiçoğlu A. (2021). Prone versus Barts “flank-free” modified supine percutaneous nephrolithotomy: A match-pair analysis. Turk. J. Med. Sci..

[19] Ozdemir H., Erbin A., Sahan M., Savun M., Cubuk A., Yazici O., Akbulut M.F., Sarilar O. (2019). Comparison of supine and prone miniaturized percutaneous nephrolithotomy in the treatment of lower pole, middle pole, and renal pelvic stones: A matched pair analysis. Int. Braz. J. Urol..

[20] Chow A.K., Ogawa S., Seigel C., Sands K.G., Vetter J., Desai A., Venkatesh R.J. (2021). Evaluation of perirenal anatomic landmarks on computed tomography to reduce the risk of thoracic complications during supracostal percutaneous nephrolithotomy. J. Endourol..

